# State-of-Charge Estimation for Lithium-Ion Batteries Using Residual Convolutional Neural Networks

**DOI:** 10.3390/s22166303

**Published:** 2022-08-22

**Authors:** Yu-Chun Wang, Nei-Chun Shao, Guan-Wen Chen, Wei-Shen Hsu, Shun-Chi Wu

**Affiliations:** Department of Engineering and System Science, National Tsing Hua University, 101, Section 2 Kuang Fu Road, Hsinchu 30013, Taiwan

**Keywords:** state-of-charge, lithium-ion battery, deep learning, residual convolutional neural networks

## Abstract

State-of-charge (SOC) is a relative quantity that describes the ratio of the remaining capacity to the present maximum available capacity. Accurate SOC estimation is essential for a battery-management system. In addition to informing the user of the expected usage until the next recharge, it is crucial for improving the utilization efficiency and service life of the battery. This study focuses on applying deep-learning techniques, and specifically convolutional residual networks, to estimate the SOC of lithium-ion batteries. By stacking the values of multiple measurable variables taken at many time instants as the model inputs, the process information for the voltage or current generation, and their interrelations, can be effectively extracted using the proposed convolutional residual blocks, and can simultaneously be exploited to regress for accurate SOCs. The performance of the proposed network model was evaluated using the data obtained from a lithium-ion battery (Panasonic NCR18650PF) under nine different driving schedules at five ambient temperatures. The experimental results demonstrated an average mean absolute error of 1.260%, and an average root-mean-square error of 0.998%. The number of floating-point operations required to complete one SOC estimation was 2.24 × 10^6^. These results indicate the efficacy and performance of the proposed approach.

## 1. Introduction

Lithium-ion (or Li-ion) batteries are rechargeable batteries with a high energy density, no memory effect, and low self-discharge [1]. They have been widely used in portable electronics and have become increasingly popular for electric vehicles. State-of-charge (SOC) is a relative quantity that describes the ratio of the remaining capacity to the present maximum available capacity of a battery. Accurate SOC estimation is crucial in a battery-management system to inform the user of the expected usage until the next recharge, and to improve the utilization efficiency and service life of the battery [2]. Various methods facilitate the estimation of the SOC for Li-ion batteries, including Coulomb-counting, open-circuit-voltage (OCV), and model-based estimation methods. 

SOC estimation in Coulomb counting is realized by integrating the charging and discharging currents over time [3,4]. Accurate current sensing and a correct initial SOC estimate are crucial for successful Coulomb counting. Moreover, the influence factors (e.g., the operating temperature) on the Coulombic efficiency should be carefully considered. The OCV, which is a nonlinear function of the SOC, is obtained through an offline OCV test at a specific ambient temperature and aging stage [4,5]. This approach is known for its computational efficiency, which is implemented as either a look-up table or an analytical expression in a battery-management system. However, the relaxation time of the Li-ion battery can be long, which limits the practicality of OCV. Moreover, for chemistries such as lithium iron phosphate [6], the SOC–OCV relationship is relatively flat; therefore, a small error in the OCV measurement could lead to a significant error in the estimated SOC. 

Model-based methods require a battery model that relates the SOC and the dynamic behaviors of the battery; thus, the SOC can be inferred from the measurable variables (e.g., voltage, current, and temperature) that characterize its dynamic behaviors under that SOC [5,7]. The advantage of this method category lies in its reliability, accuracy, and universal validity. However, developing a model that can adequately describe a battery requires laborious experiments and extensive battery research by domain experts [8]. Moreover, the model parameters vary over the lifetime of a battery, and accurate estimations can only be attained in the laboratory or achieved by using sophisticated approaches [7]. Finally, many external uncertainties in the ambient environment may alter the internal electrochemical behavior of the battery. Formulating a model that considers all these factors for accurate SOC estimation is not easy because of the complex nonlinearity and time variability of the system [8]. Examples of model-based methods include the Luenberger observer [9], adaptive nonlinear observer [10], extended Kalman filter (EKF) [11], and unscented Kalman filter (UKF) [12]. The Luenberger observer is widely used for linear deterministic dynamic systems due to its simplicity. However, its SOC estimates may deviate from actual values due to the nonlinearity of the OCV over a wide SOC range [9]. The adaptive nonlinear observer is designed to compensate for the nonlinearity and it can attain better SOC-estimation accuracy [10]. The EKF is an alternative to address the issue based on the principle of the linearization of the nonlinear function that uses partial derivatives and first-order Taylor series expansion [11]. Considering that high orders are ignored in the EKF, the UKF may also be adopted in a highly nonlinear system if needed [12].

Relying on computational intelligence techniques to perform SOC estimation, data-driven methods are gaining significant attention. The measurable variables of a battery vary, where the degree of change depends on the SOC level. Different profiles among these variables are exhibited under different SOCs. The methods aim to establish connections between these profiles and their corresponding SOCs without the need for prior information about the internal characteristics and chemical reactions of the battery [3,8]. Examples of this category include works based on support vector machines [13], artificial neural networks (ANNs) [14], and functional link neural networks [15]. SOC estimation in these methods is considered as a regression problem, and efforts are made to evaluate the efficacy of different methods to obtain a “hyper-plane” that can reasonably fit the “data” (i.e., SOC, voltage, current, and temperature). Because the relationships between the measurable variables and SOC cannot be adequately described using a linear model, kernel functions that map the data to a high-dimensional space are required. However, estimating the regression coefficients of higher-order models is difficult. Among these methods, neural networks have been shown to have suitable nonlinear-function-approximation capabilities [14]. 

Deep learning is a branch of machine learning based on ANNs [16]. It has been applied in many fields, including SOC estimation. Some of the latest deep-learning models used for SOC estimation include the deep feedforward neural network (DNN)-based [17] and gated recurrent unit (GRU)-based network models [18,19]. The advantages of a DNN model include its capability of directly mapping the measurable variables to the SOC and self-learning its weights through learning algorithms, such as gradient descent. This is markedly different from techniques such as equivalent-circuit and electrochemical models, which require much time to hand-engineer and parameterize [17]. The capacitive effect in the Li-ion battery causes the previous states of the battery to influence its present state [14], and including the past-state information while performing SOC estimation can be beneficial. However, the DNN model lacks this ability. A GRU is an advanced version of the recurrent-neural-network (RNN) architecture. Although an RNN can use the internal state to learn time dependencies from sequential data [19], it has been revealed to be unable to capture long-term dependencies due to the so-called vanishing gradient problem [18]. On the contrary, a GRU is not only able to capture long-term sequential dependencies, but it is also robust to the vanishing gradient problem [19]. These network models have superior capabilities in timeseries analysis and may be able to use past information for long periods. However, to attain a high estimation accuracy, a highly complicated model may be required (e.g., the GRU-based network of [19]). Moreover, these models focus on exploring the information contained in the measurable variables from a temporal aspect (e.g., the process information for voltage or current generation), which leads to the ignorance of their interactions. A convolutional neural network (CNN) is a deep-learning-network model initially designed to work with an image. However, its application to multivariate data timeseries analysis for sensor-reading reconstruction and SOC estimation have also been reported [20,21].

Unlike the existing approaches, this study focuses on applying deep-learning techniques, and specifically convolutional residual networks, to estimate the SOC of a Li-ion battery. We aim to propose a network model that exploits the process information and the interrelations among the measurable variables to achieve accurate SOC estimation. Moreover, the model needs to be compact to be applicable in real-time applications. Because of the capacitive resistance in a battery, the past currents and voltages of a Li-ion battery affect its present state [14]. Thus, we include the values of multiple measurable variables taken at many time instants as the model inputs while performing the SOC estimation. Before proceeding to the SOC estimation, the data of different measurable variables are stacked as a two-dimensional data block that may be regarded as an “image”. Although including measurable variables for SOC estimation, such as the voltage, current, and temperature taken at many time instants, can provide different insights into the task, the large volumes of data unquestionably pose challenges in revealing the discriminant information therein. To prevent an estimation model from being overwhelmed by the massive amount of data, convolution layers are included. A convolution layer is known for its superior ability to extract discriminant information from an image. Through the filters therein, the process information for the voltage or current generation (usually explored by the ANN- and GRU-based networks) and their interrelations can be extracted and exploited to regress for accurate SOCs. To emphasize, although a convolutional neural network is frequently used for image classification, its application to timeseries analysis is straightforward in this study due to the way we treat the acquired data (i.e., stacking them as a two-dimensional data block). After the convolution layers, several fully connected layers are utilized to regress the obtained discriminant information for the SOC. In addition, we include several shortcut connections in the proposed model, which perform identity mapping to prevent the accuracy saturation problem when the network goes deeper [22]. These connections ensure that the higher layer performs at least as well as the lower layer, if not better. They also provide the benefit of not adding any extra parameters or computational complexity.

The remainder of this paper is organized as follows. Two existing deep-learning-based approaches for SOC estimation are reviewed in Section 2. The proposed network model is described in detail in Section 3. The datasets used for the performance evaluation and the results from the detailed experiments are provided in Section 4 and Section 5, respectively. Finally, the conclusions are presented in Section 6.

## 2. Existing Deep-Learning-Based Methods for SOC Estimation

In this section, we review two existing SOC-estimation network models. We emphasize their architectures, data-normalization methods, input data, and loss functions.

### 2.1. Deep Neural Network (DNN)

The DNN of Chemali et al. [17] comprises seven fully connected layers with complete connections to all activations in the previous layer. The first layer (i.e., the input layer) contains four neurons to accommodate an input-data vector of the size 4 × 1, and the last layer has one single neuron for the regression of the SOC. The number of neurons in the layers in between is 32. The input-data vector is composed of the voltage, temperature, average current, and average voltage obtained at time (*t*) without additional normalization: $x_{t}={\left[V_{t},\;T_{t},\;I_{t}^{avg},\;V_{t}^{avg}\;\right]}^{T}$. The average current and voltage at time (*t*) are calculated using the currents and voltages taken at the last 400 time instants. The activation function of the last layer is the identity function, and the rectified linear unit (ReLU) is used in the remaining layers. The ReLU function returns 0 if it receives any negative input; otherwise, it returns the input itself. Compared with other activation functions, it reduces the likelihood of the vanishing gradient problem. Moreover, the constant gradient of the ReLU leads to faster learning [23,24].

The weights in different layers are determined by minimizing the loss function:

(1) $$L=e_{max}^{2}+\frac{1}{n}\sum_{t=0}^{n}e_{t}^{2},$$

where *n* is the length of the data sequence used for training, and $e_{t}$ represents the estimation error, defined as $e_{t}=SOC_{t}-SOC_{t}^{*}$, with $SOC_{t}$ and $SOC_{t}^{*}$ being the actual and estimated SOC at time (*t*), respectively. $e_{max}$ is the maximum estimation error. A complete training epoch has one forward pass and one backward pass. The forward pass starts when the training-data vectors are fed to the network model, and it ends when the corresponding SOCs are estimated, and the loss function is evaluated. The backward pass represents the process of sending the loss-function value backward through the network to update the weights, which is accomplished by the adaptive-moment-estimation (Adam) optimizer. The training process is stopped when the maximum number of epochs is met.

### 2.2. Gated Recurrent Neural Network

The capacitive effect in the Li-ion battery causes the previous states of the battery to influence its present state [14], and including the past-state information while performing SOC estimation can be beneficial. However, the DNN model does not have this ability. To address this issue, GRU-based network models have been proposed [18,19]. A GRU is an advanced version of the RNN architecture. By introducing an update gate to determine the proportion of the candidate state to be accepted, and a reset gate to restrict the impact of the previous hidden state on the candidate state, capturing the long-term dependency in a data series becomes possible [25].

We describe the GRU network model proposed in Li et al. [19] as follows: Following the input layer of three neurons, one GRU layer is added to learn the temporal dependency among the input data. Thereafter, a fully connected layer is followed before entering the regression output layer for the SOC estimation. Specifically, the full connection layer transforms multiple outputs of the GRU layer into a single SOC estimate. The number of units in the GRU layer is 1000, and the number of neurons in the fully connected layers is 50. The network parameters are optimized using the Adam optimizer. The input-data vector is composed of the voltage, current, and temperature of the battery at time (*t*): $x_{t}={\left[V_{t},\;I_{t},\;T_{t}\;\right]}^{T}$, and more than one data vector (e.g., 1000 is suggested in [19]) can be used to estimate the SOC at a given time. To avoid the influence of magnitude imbalance on the performance of the SOC estimation, the data of different measurable variables are normalized before further application using:

(2) $$x_{t}^{normalized}=\frac{2\left(x_{t}-x_{min}\right)}{\left(x_{max}-x_{min}\right)}-1,$$

where $x_{t}$ is the value of a specific measurable variable (e.g., voltage) acquired at time (*t*), with $x_{max}$ and $x_{min}$ being its possible maximum and minimum values obtained from the training data, respectively. Finally, the loss function used in this model is the mean absolute error (MAE), defined as:

(3) $$L=\frac{1}{n}\sum_{t=0}^{n}\left|e_{t}\right|.$$

The training process stops when the maximum number of epochs is satisfied.

## 3. Proposed Network Model for SOC Estimation 

To exploit the temporal information and the interrelations in the multiple measurable variables of the battery for SOC estimation, we stack their values obtained at *n* time instants as a matrix: $X_{t}=\left[x_{t},\;x_{t-1},\;\cdots ,\;x_{t-n+1}\right]$, with $x_{t}={\left[V_{t},\;I_{t},\;T_{t}\;\right]}^{T}$, which is the input to our proposed network model. We will discuss how the parameter *n* is determined in Section 5.2. Before constructing the $X_{t}$, the data of the different measurable variables are normalized using [26]:

(4) $$x_{t}^{normalized}=\frac{x_{t}-x_{min}}{x_{max}-x_{min}},$$

where $x_{t}$ is also the value of a specific measurable variable acquired at time (*t*), and $x_{max}$ and $x_{min}$ are its possible maximum and minimum values, respectively. The idea of treating multisensor data as an “image” (i.e., a two-dimensional data block) is crucial to our discussion. The main building blocks of this network are first introduced, before proceeding to the details of the proposed network model.

### 3.1. CNNs and Residual Blocks

**Figure 1:**
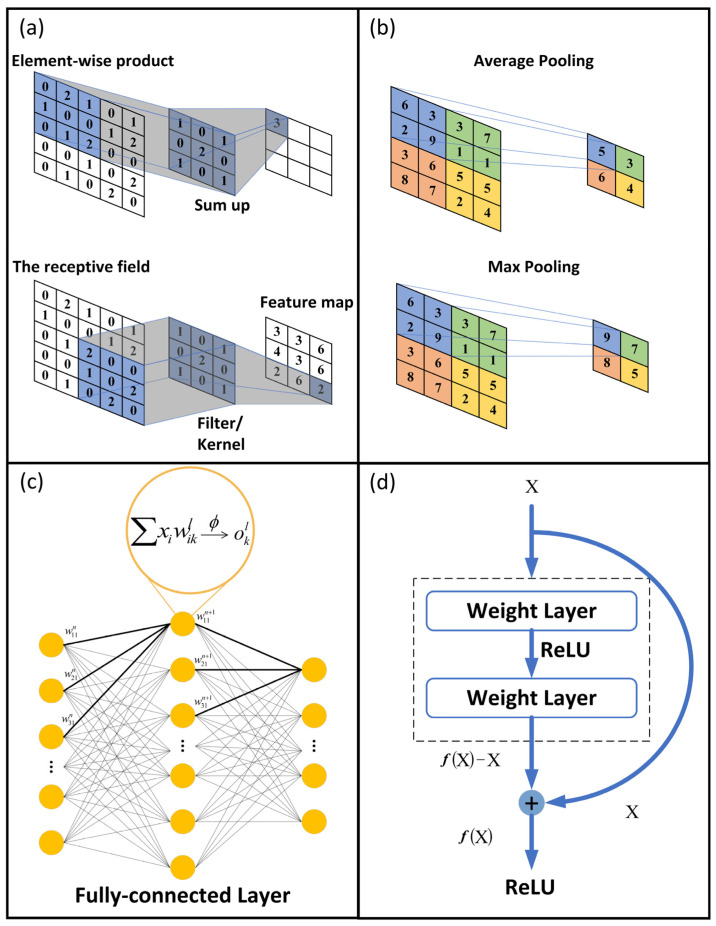
Composition blocks of a residual convolutional neural network. (**a**) A convolution layer and a convolution operation using a 3 × 3 filter/kernel. (**b**) A pooling layer and two typical pooling operations using a 2 × 2 filter. (**c**) A series of three fully connected layers having different numbers of neurons. (**d**) A conceptual residual block.

A CNN is a deep-learning network with layers of different characteristics [16,27]. A convolution layer performs a dot product between two matrices (i.e., the convolution operation), as shown in Figure 1a: one is a set of learnable parameters, known as a kernel or filter, and the other is a portion of the input data [28]. This filter is applied across the entire input space to obtain a feature map, and an arbitrary number of filters can be utilized to extract the input-data characteristics from different aspects. A pooling layer is used to downsample the feature maps obtained above [29]. This involves the extraction of non-overlapping patches (e.g., having a size of 2 × 2) from a feature map and computing the average or maximum of the features therein as the output, as shown in Figure 1b. The downsampled feature maps are less sensitive to changes in the positions of the features in the original maps. Neurons in a fully connected layer have connections to all activations in the previous layer, as shown in Figure 1c. These layers consider the results of the convolution/pooling process to reach a classification decision or regression value. The feature map must be transformed into a one-dimensional array of numbers or vectors before connecting to a fully connected layer. This process is referred to as “flatten”. Finally, the activation function applied to the output layer is determined by the task to be performed. The softmax function is used for classification, and the identity function (i.e., *f*(*x*) = *x*) is adopted for regression [30]. 

A deeper network may fail to perform better than its shallow counterpart, and it is better to work backward when this happens. However, if we can let a layer stack coming after a shallow subnetwork inside a deep model do nothing (i.e., performing an identity mapping), the performance and training error of the deeper model should be comparable with those of the shallow subnetwork [22]. This idea of containing an identity function as one of the elements in every additional layer stack lies at the core of a residual block. 

The residual block shown in Figure 1d is the building block of the residual neural network. It comprises a stack of layers set in such a way that its input is taken and added to the output of the block before passing the activation function. The bypass connection is referred to as a shortcut connection. If we denote the input of a residual block by X (e.g., an image or a feature map) and assume that the desired mapping to learn is *f*(X), then the portion of the residual block enclosed by the dashed lines must directly learn the mapping *f*(X) when the shortcut connection is absent. However, with the help of the shortcut connection, it only needs to learn the residual mapping (i.e., *f*(X) → X). If the identity mapping (i.e., *f*(X) = X) is the desired mapping, then it only needs to let the weights and biases of the layers within the dashed-line box be zero. Letting the layer stack fit a residual mapping is more accessible than directly fitting the desired underlying mapping. As a result, an effective deep neural network can be trained using the residual blocks [31].

### 3.2. Architecture of the Proposed Network

**Figure 2:**
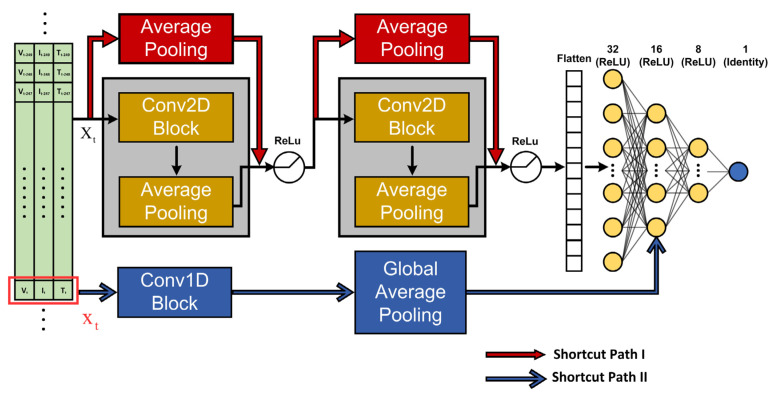
The proposed residual network model for SOC estimation. The inputs to this model are the data matrix ($X_{t}$) and the instantaneous measurement vector ($x_{t}$).

The proposed network model for SOC estimation is shown in Figure 2. After the input-data matrix ($X_{t}$) is fed in, it immediately passes through two residual blocks. Each block has one convolution layer, one average pooling layer, and one shortcut connection. The shortcut connection adds the respective input to a residual block to its output feature maps after an average pooling operation with a 1 × 2 filter. The convolution layer in each of the residual blocks has 16 3 × 3 filters. We will discuss how the number of filters is determined in Section 5.2. The size of the filter used in the two average pooling layers is 1 × 2. All filters in the convolution and pooling layers are applied with a stride of one. To regress the results from the residual blocks for the SOC, three fully connected layers are added in series, without any intervening layer, before reaching the single output neuron. The numbers of neurons in the three fully connected layers are 32, 16, and 8. Motivated by the Kalman-filter-based approach, a shortcut connection is added to directly present the current measurement vector ($x_{t}$) of the battery to the second fully connected layer for the SOC estimation. Before the $x_{t}$ is added to the output of the second fully connected layer, it goes through a convolution operation incorporating 16 3 × 1 filters and a global pooling operation. The global average pooling operation flattens the feature maps by taking the average of each feature map and stacking them as a data vector for the following application. All the trainable layers are followed by the ReLU activation function, except for the output layer, where the identity activation function is used. Finally, the MAE given in (3) is used as the loss function. 

The network parameters are optimized using the Adam optimizer, and early stopping is used to halt the training to prevent overfitting [32]. Finding an optimal network model (i.e., searching for the optimal weights for a network model) for SOC estimation can be formulated as an optimization problem. Once the loss function (e.g., the MAE given in (3)) is determined, the problem can be solved by an appropriate optimization method. Adam is a method for stochastic optimization [33,34]. It combines the advantages of adaptive gradients and root-mean-square propagation (RMSProp). Instead of using the entire dataset to calculate the actual gradient, this algorithm uses a randomly selected data subset to create a stochastic approximation. The learning rate for gradient descent controls the amount that the weights are updated. If it is low, then training will progress slowly; if it is too high, then this leads to undesirable divergent behavior. Moreover, rather than adapting the learning rate based on the average first moment, as in RMSProp, Adam also uses the average of the second moments of the gradients. Specifically, the algorithm calculates an exponential moving average of the gradients and squared gradients, and the parameters $\beta_{1}$ and $\beta_{2}$ control the decay rates of these moving averages. These are the key hyperparameters to set in the Adam optimizer [33]. 

We additionally include the data vectors taken previously for SOC estimation because the past voltages, currents, and temperatures will affect the current SOC owing to the capacitive effect in the battery [14]. Discarding the measurable data taken previously could lead to a loss of information. However, including a large volume of data for SOC estimation unquestionably poses challenges in revealing discriminant information for the task, and a scheme to prevent the proposed model from being overwhelmed by a massive amount of data must be considered. Notably, a filter in the convolution layer is applied across the entire input space. It moves from left to right with a particular stride until it parses the complete width. This is similar to the multivariate autoregressive (MVAR) model. An MVAR model formed by a weighted linear sum of the input-data vectors can predict future data vectors [35,36]. The learnable parameters in the filter are the required predictor coefficients in the MVAR model. Different filters can lead to different MVAR models. The pooling operation mainly adopted in this study extracts patches of a 1 × 2 size from an input feature map, and it uses the mean of these extracted features as their respective output (i.e., average pooling shown in Figure 1b). Note that the pooling is applied in the row direction (i.e., time indices) of the X, which allows the noisy activations to be discarded. As the selected measurable variables are not physically swapped while constructing the input-data matrix ($X_{t}$), a shift in the column direction of the $X_{t}$ is not expected to occur. Thus, a size of one is utilized. Unlike in the convolution layers, there are no learnable parameters in the pooling layers.

## 4. Battery Datasets

The Li-ion battery dataset provided by the research group at the University of Wisconsin–Madison [37] was utilized to evaluate the performance of the proposed network model. The dataset contains data from a 2.9 Ah nickel cobalt aluminum chemistry Li-ion battery (Panasonic NCR18650PF), tested with a 25 amp 18 V Digatron Firing Circuits Universal Battery Tester (Digatron Firing Circuits, Shelton, CT, USA) channel placed in an eight-cubic-foot thermal chamber. The cell was cycled according to nine different driving schedules: US06, HWFET, UDDS, LA92, neural network (NN), and Cycles 1 to 4, under ambient temperatures of 25, 10, 0, −10, and −20 °C. US06 is developed to reflect aggressive, high-speed, and high-acceleration driving behaviors. The highway fuel economy test (HWFET) cycle is a driving schedule for determining the highway fuel economy. The urban dynamometer driving schedule (UDDS) represents urban driving conditions. These are the driving schedules used by the United States Environmental Protection Agency (EPA, Washington, DC, USA) for vehicle emissions and fuel economy testing. Developed by the California Air Resources Board, LA92 is a dynamometer driving schedule for light-duty vehicles, but it is a more aggressive driving cycle than the EPA federal test procedure (FTP-75) [38]. The NN driving schedule consists of US06 and LA92, and it is designed to have different dynamics that are helpful in training neural networks. Finally, Cycles 1 to 4 are formed by randomly mixing the US06, HWFET, UDDS, LA92, and NN drive schedules. This dataset covers a wide range of variability, making the performance evaluation of the proposed network model realistic. Finally, measurable variables, such as the voltage, current, capacity, battery temperature, and chamber temperature, were recorded approximately every 0.1 s, with a slight variance in the sampling rate. Further detailed experimental descriptions can be found in the accompanying “ReadMe” file [37].

## 5. Experiments and Discussion

### 5.1. Experimental Details

As mentioned above, US06, HWFET, UDDS, and LA92 are the driving schedules used for vehicle emissions and fuel economy testing. We used their corresponding data for the model training. To obtain a single model applicable to SOC estimation under different ambient temperatures, all the data were simultaneously included for model training, regardless of the ambient temperatures under which they were obtained. Furthermore, because the NN driving schedule was specially designed to help train neural networks, its related data were used as the validation set. Finally, the data of the remaining four driving schedules were retained for model testing. The data corresponding to different driving schedules used for model training, validation, and testing are summarized in Table 1. Before proceeding, we interpolated all the data and resampled them at a sampling rate of 1 Hz. The maximum and minimum values of the measurable variables used for the data normalization are listed in Table 2. In addition to the proposed network model, we also implemented the network models of Chemali et al. [17] and Li et al. [19] for comparison. These two network models are hereafter referred to as the DNN- and GRU-based models, respectively. The MAE and root-mean-square error (RMSE) used in most studies [19,21,39] were adopted to quantify the prediction performance. The RMSE is defined as follows:

(5) $$RMSE=\sqrt{\frac{1}{n}\sum_{t=0}^{n}\left|e_{t}\right|}.$$

Finally, all the network models were trained on TensorFlow 1.13.1 (Google, Mountain View, CL, USA) with the CUDA 9.2 Toolkit (NVIDIA, Santa Clara, CA, USA) and cuDNN v7.6.0 (NVIDIA, Santa Clara, CA, USA) on an ASUS ESC8000 G4 server (ASUS, Taipei, Taiwan) with an Intel Xeon CPU (Intel, Santa Clara, CA, USA), GeForce RTX 2080 Ti (NVIDIA, Santa Clara, CA, USA), and 192 GB RAM. The required hyperparameters used for training the DNN- and GRU-based models were set following [17] and [19], with a learning rate of 10^−4^, and decay rates of $\beta_{1}$ = 0.9 and $\beta_{2}$ = 0.999, respectively. The maximum numbers of epochs were 10,000 and 100, respectively. As for the proposed network model, the decay rates were the same, but the learning rate was 10^−3^. They are the default settings suggested in the original paper on the Adam optimizer [33]. 

### 5.2. Results and Discussion

**Figure 3:**
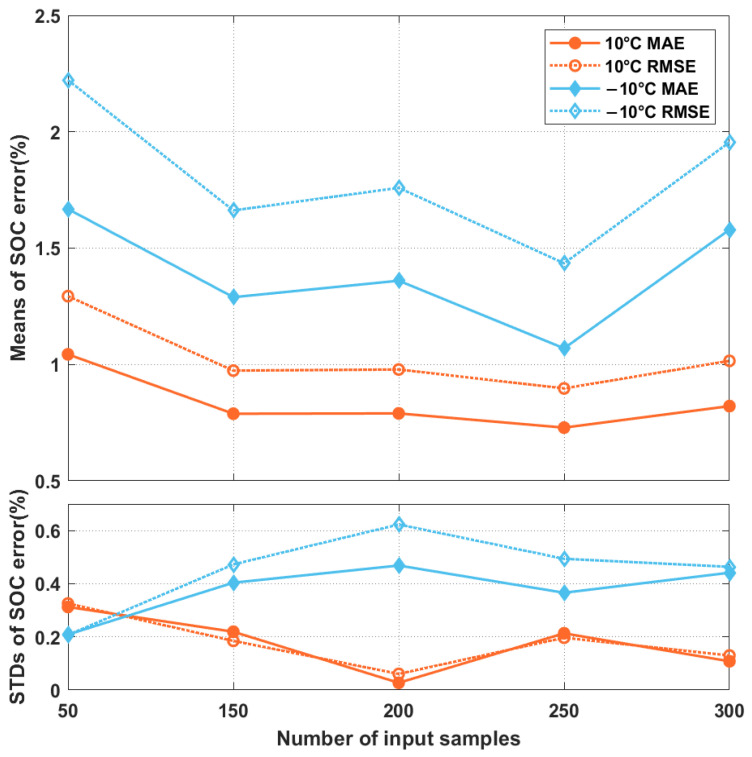
The mean MAEs and RMSEs of the proposed network model while using different numbers of measurement vectors for the $X_{t}$ construction and the corresponding standard deviations (STDs). The results were obtained by estimating the SOCs of Cycles 1 to 4.

Although including the past-state information of the battery in the proposed network model can benefit the SOC estimation, the amount of information to be used in terms of the number of measurement vectors is unclear. Thus, in the first experiment, we studied how the number of measurement vectors used to form the input matrix (**X***_t_*) affects the accuracy of the SOC estimation. Figure 3 shows the mean MAEs and RMSEs, as well as their standard deviations (STDs), obtained by the proposed network model while estimating the SOCs of the driving schedules Cycles 1 to 4 at ambient temperatures of 10 °C and −10 °C. We varied the number from 50 to 300, and we set the number of filters in the two residual blocks to 16. As shown in Figure 3, the MAEs and RMSEs varied under different numbers of measurement vectors. Gradual decreases in the MAEs and RMSEs were observed when the number increased from 50 to 250. When it exceeded 250, the MAEs and RMSEs started to increase. The trends of the MAEs and RMSEs were similar under different ambient temperatures. Although the capacitive resistance in the battery causes a battery state to affect the battery’s future state, this influence is temporary. Moreover, including excessive uncorrelated state information cannot enhance the SOC estimation and may instead degrade the performance owing to an increase in the model complexity.

**Figure 4:**
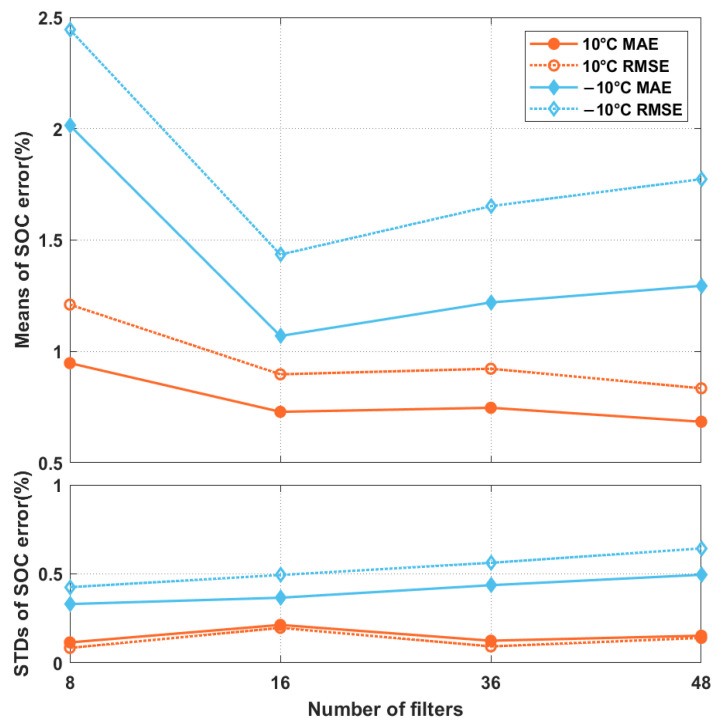
The mean MAEs and RMSEs, and their STDs, of the proposed network model while different numbers of filters were used in the convolution layers of the two residual blocks.

In the second experiment, we studied the influence of the number of filters in the convolution layers of the two residual blocks on the SOC-estimation accuracy. Different filters allow different input-data characteristics to be revealed. However, the larger the number of filters, the more learnable the parameters will be. This requires more data to train the network. Moreover, because many filters exist, some may learn characteristics that are not important for SOC estimation. Filter-pruning methods that remove unimportant filters in a network model are not uncommon in the literature [40]. To determine a suitable number of filters to use, we varied the filter number from 8 to 48, and the number of measurement vectors for the $X_{t}$ formation was set to 250. As shown in Figure 4, with few filters, the mean MAEs and RMSEs were large, regardless of the ambient temperature. As the number of filters increased, the errors first decreased and then increased. The MAE and RMSE were small when the number of filters was 16. Because of the satisfactory estimation errors achieved, for the experiments discussed hereafter, we implemented the proposed scheme with 250 measurement vectors for the $X_{t}$ formation, and 16 filters in the residual blocks.

**Figure 5:**
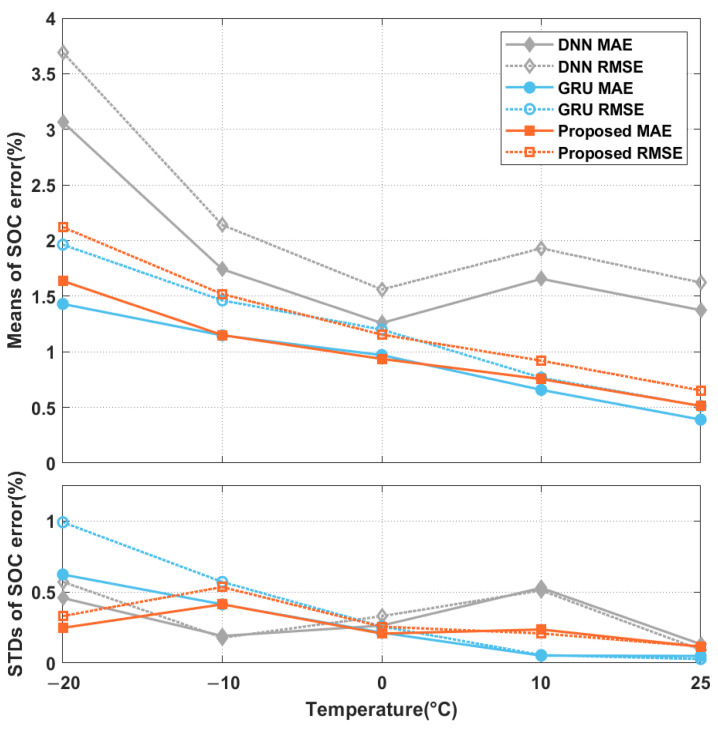
The mean MAEs and RMSEs, and their STDs, of three network models for SOC estimation under different ambient temperatures.

**Figure 6:**
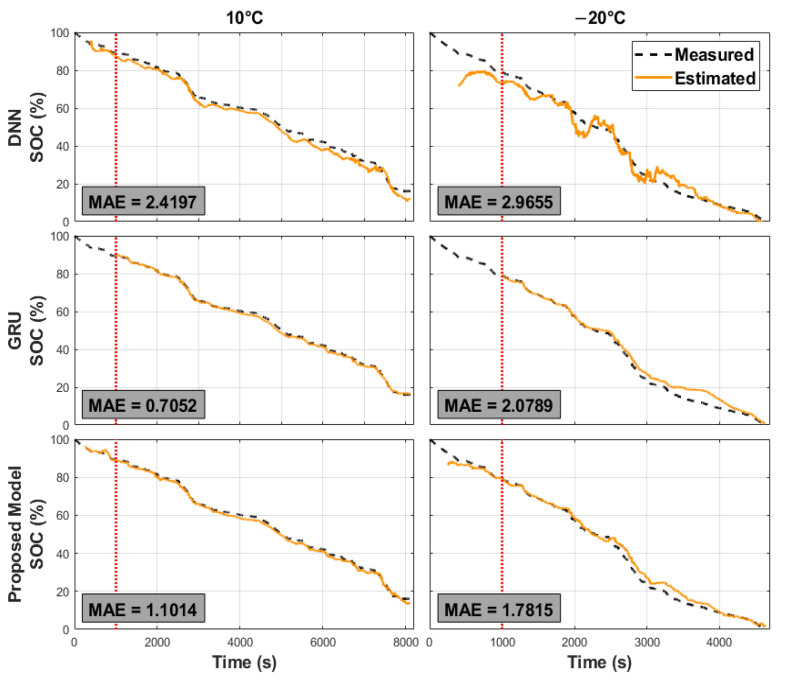
Cycle 2 SOC-estimation results of three different network models at two ambient temperatures: 10 °C and −20°C. Top row: DNN-based model. Middle row: GRU-based model. Bottom row: the proposed model.

**Figure 7:**
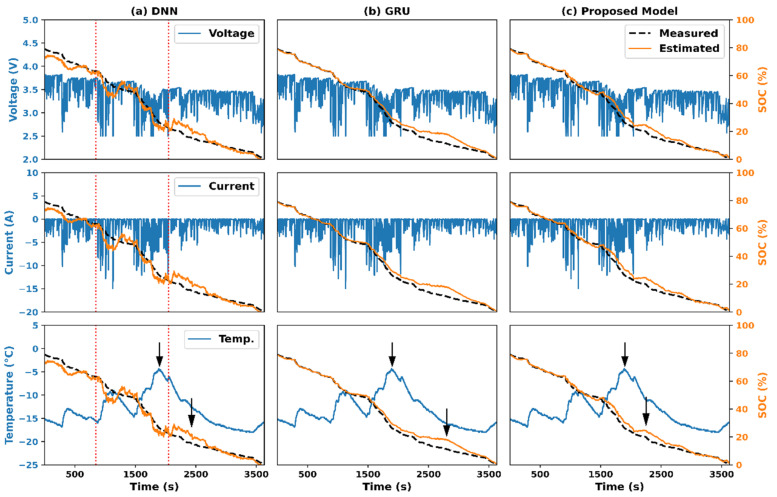
Cycle 2 SOC-estimation results (solid orange lines) using: (**a**) DNN-based model, (**b**) GRU-based model, and (**c**) the proposed model. The corresponding voltages, currents, and temperatures are also provided. The ambient temperature was −20 °C. The area between the two red dotted lines indicates the measurable variable varied significantly, and the black arrows indicate the evident discrepancies between the three approaches’ actual and estimated SOC curves when a sudden increase in the battery temperature.

In the third experiment, we compared the performances of the three approaches. The MAEs and RMSEs of the different approaches averaged over the driving schedule Cycles 1 to 4, and their STDs, are shown in Figure 5. The estimation errors increased as the ambient temperature decreased. The DNN-based approach performed poorly because of the simple network architecture adopted and the inability to exploit the past-state information of the battery for SOC estimation. Furthermore, the estimated SOCs fluctuated, as shown in Figure 6. In many applications, it is desirable that the SOC estimation evolves smoothly around the actual values so that the residual-range prediction will not suddenly increase or decrease and confuse the user [14]. Thus, an additional postprocessing scheme (e.g., the UKF) is required to cooperate with one such network model to provide smooth SOC estimates with sufficient accuracy. By further looking at the times when the SOC estimates fluctuated a lot, we noticed that the measurable variables, mainly the voltages and currents, varied significantly, as shown in Figure 7a, between the two dotted lines. This indicates that the SOC estimated by the DNN-based approach was sensitive to the variations in the input data, although a moving average (MA) was applied while preparing the model inputs. These deficiencies could be addressed using the GRU-based and proposed network models, as shown in Figure 6 and Figure 7, where the actual and estimated SOC curves were close to each other without the need for any additional postprocessing scheme. The smoothing ability in the GRU-based model could be because the GRU units can not only perform an MA on the lagged inputs, but can also regress on their own lagged outputs, which is a vital characteristic of an autoregressive (AR) model. Noise reduction in data timeseries using ARMA filtering is not uncommon in the literature [41]. As for the proposed scheme, this could be owing to the ability of the average-pooling layers to discard the noisy activations while performing dimensionality reduction. Furthermore, the filters in the convolution layers can reduce noise [42]. Finally, the poor performance of the three approaches when the ambient temperature was low could be due to the significant disparity between the measured surface temperature of the battery and its internal temperature [16]. As shown in Figure 7, a sudden increase in the battery temperature was followed by evident discrepancies between the three approaches’ actual and estimated SOC curves, as indicated by the black arrows. The estimation errors of the three implemented approaches under different driving schedules and ambient temperatures are summarized in Table 3. The MAEs and RMSEs of the GRU-based and proposed models were comparable, with approximately half the errors of the DNN-based model. Their maximum estimation errors are listed in Table 3. The maximum estimation errors of the proposed and GRU-based models were 8.673% and 6.870%, respectively, which were lower than that of the DNN-based model (i.e., 14.062%).

In the final experiment, we analyzed the computational complexity of the three network models to provide one SOC estimate. The computational complexity in terms of the number of floating-point operations (flops) was calculated using the method in [43]. The computational cost of the DNN-based model was 4400 flops. Under the same settings, 4.33 × 10^8^ and 2.24 × 10^6^ flops were required for the GRU-based and proposed approaches, respectively. Despite its low computational complexity, the SOC estimation using the DNN-based model was error-prone, as mentioned previously. Although the GRU-based model attained better estimation accuracy, its computational complexity was of substantial concern. However, this concern was largely reduced in the proposed model. Furthermore, the computational complexity of the proposed network model could be further reduced using a suitable filter-pruning scheme [37], if required. Lastly, we calculated the average times required to perform one SOC estimation in our platform by averaging the times needed to complete 29,640 SOC estimations (i.e., four testing cycles, with each of them having 7410 samples). The results were 4.62 × 10^−5^ s, 4.64 × 10^−3^ s, and 1.07 × 10^−4^ s for the DNN-based, GRU-based, and proposed models, respectively. It was found that the run times required by these models on our computing platform were less than 1 s; therefore, they all could provide a new SOC estimate before new values of the measurable variables arrived.

## 6. Conclusions

This paper presents a convolutional residual network to estimate the SOC of Li-ion batteries. By stacking the values of multiple measurable variables taken at many time instants as the model inputs, the process information for the voltage or current generation and their interrelations can be effectively extracted by the proposed convolutional residual blocks and can simultaneously be exploited to regress for accurate SOCs. In addition, including the shortcut connections that allow the higher layer to perform at least as well as the lower layer makes the entire network model compact. The performance of the proposed network model was evaluated using the data obtained from a Panasonic NCR18650PF Li-ion battery. For a testing scenario involving four mixing driving schedules, the proposed model could accurately estimate their SOCs at different ambient temperatures, with a mean MAE of 0.998%, and a mean RMSE of 1.260%. Moreover, the number of flops required to complete one SOC estimation was only 2.24 × 10^6^. All these results illustrate the efficacy of the proposed network model.

## Figures and Tables

**Table 1:** The Li-ion battery datasets [37] used to evaluate different SOC-estimation network models. The data of different driving schedules for model training, validation, and testing are shown as indicated.

Temp.	UDDS	LA92	US06	HWFET	NN	Cycle 1	Cycle 2	Cycle 3	Cycle 4
25 °C	Train	Train	Train	Train	Validation	Test	Test	Test	Test
10 °C	Train	Train	Train	Train	Validation	Test	Test	Test	Test
0 °C	Train	Train	Train	Train	Validation	Test	Test	Test	Test
−10 °C	Train	Train	Train	Train	Validation	Test	Test	Test	Test
−20 °C	Train	Train	Train	Train	Validation	Test	Test	Test	Test

**Table 2:** The maximum and minimum values of different measurable variables used for data normalization in this study.

	Voltage (V)	Current (A)	Temperature (°C)
Max	4.4	10	30
Min	2.5	−10	−25

**Table 3:** SOC-estimation errors (MAEs, RMSEs, and maximum errors) of the three network models under different driving schedules and ambient temperatures.

Temp.	Operating Condition	MAE (%)			RMSE (%)			MAX (%)		
		DNN	GRU	Proposed	DNN	GRU	Proposed	DNN	GRU	Proposed
25 °C	Cycle 1	1.543	0.460	0.673	1.734	0.549	0.846	4.072	1.557	1.817
	Cycle 2	1.281	0.386	0.410	1.557	0.520	0.515	5.196	1.535	2.106
	Cycle 3	1.416	0.376	0.515	1.662	0.487	0.644	4.350	1.262	1.709
	Cycle 4	1.258	0.342	0.457	1.535	0497	0.604	4.664	1.770	2.030
10 °C	Cycle 1	1.438	0.696	0.689	1.708	0.774	0.853	5.364	1.448	2.890
	Cycle 2	2.420	0.705	1.101	2.677	0.842	1.179	5.792	1.859	2.828
	Cycle 3	1.553	0.635	0.580	1.807	0.738	0.720	4.539	1.525	2.214
	Cycle 4	1.214	0.596	0.649	1.532	0.714	0.838	4.798	2.001	2.780
0 °C	Cycle 1	1.327	1.174	1.107	1.572	1.497	1.348	4.456	3.171	3.330
	Cycle 2	1.365	1.135	1.120	1.704	1.286	1.267	4.828	2.210	2.619
	Cycle 3	0.874	0.804	0.792	1.101	1.130	0.925	3.744	2.635	2.294
	Cycle 4	1.469	0.769	0.725	1.865	0.888	0.853	6.085	2.147	2.445
−10 °C	Cycle 1	1.684	1.633	1.739	2.177	2.206	2.157	6.765	5.162	6.356
	Cycle 2	1.670	1.347	1.112	2.018	1.609	1.349	5.311	2.982	3.653
	Cycle 3	1.595	0.788	0.951	1.990	1.015	1.132	6.332	2.651	3.061
	Cycle 4	2.022	0.823	0.800	2.377	1.017	1.101	6.444	1.986	3.822
−20 °C	Cycle 1	3.557	1.126	1.826	4.195	1.303	2.887	11.677	3.886	8.673
	Cycle 2	2.966	2.079	1.782	3.673	2.904	2.336	10.175	6.870	6.012
	Cycle 3	2.483	0.716	1.282	2.899	0.936	1.621	7.720	2.980	4.553
	Cycle 4	3.255	1.800	1.659	3.999	2.711	2.028	14.062	5.854	5.956
Average		1.820	0.920	0.998	2.189	1.181	1.260	6.319	2.775	3.557

## Data Availability

Battery tests were performed at the Wisconsin Energy Institute at the University of Wisconsin–Madison (https://doi.org/10.17632/wykht8y7tg.1, accessed on 11 July 2022).
